# A paean to Arnie Levine on the occasion of his 80th birthday

**DOI:** 10.1093/jmcb/mjz066

**Published:** 2019-08-15

**Authors:** Philip W Hinds

**Affiliations:** Department of Developmental, Molecular and Chemical Biology, Tufts University School of Medicine, Boston, MA 02111, USA

It is an honor and a privilege to have received PhD-level training in the molecular basis of tumorigenesis from Arnie Levine and to have received advice and support from him as a colleague in the decades that followed. I and numerous others who have been directly and indirectly intellectually nurtured by Arnie owe much of our success in biomedical research to his selfless sharing of his keen scientific insight, and the field of cancer therapeutics is enriched overall by the contributions his lab and ‘progeny’ have made over the course of his career. I first met Arnie in the fall of 1985 as a first-year PhD candidate at Princeton. As a wide-eyed country boy (more or less) with newly minted BS and MS degrees in Biochemistry from the University of Maine, I found myself in quite a different world on Princeton’s verdant campus and shortly thereafter in the gleaming halls of the brand-new Lewis Thomas Laboratories (LTL), the building of which was undoubtedly a satisfying accomplishment for Arnie as the Chair of Molecular Biology. Charged with finding my first rotation laboratory, and not without considerable trepidation, I approached Arnie and others about my options and was immediately taken with Arnie’s warmth and excitement over the research in his lab. This led to my enthusiastic request to perform my rotation with his team (and this choice was perhaps also influenced by the fact that Mike Cole’s and Jim Broach’s labs were oversubscribed), even though I was at the time rather unsure of what a laboratory rotation really entailed.

When I started this rotation, Arnie’s lab focused predominantly on studies of EBV and of SV40 T antigen. A small offshoot of the T antigen world focused on the mysteries of the T antigen-associated p53 protein, in 1985 appreciated as a likely mediator of T antigen’s oncogenic functions, but with relatively few data to support how this might be. Arnie’s enthusiasm over the concept that a highly evolved, tumorigenic viral protein such as T antigen would be loath to dally with a cellular protein unless that protein had great significance to viral function in replication and/or cellular transformation was infectious(!), and I happily agreed to try to help out with this research direction. A particular conundrum had arisen concomitant with my arrival in the Levine lab, in that other groups had used cloned p53 cDNAs and genomic fragments to transform primary rat cells (most notably in two Nature papers published in 1984, one resulting from a collaboration between Varda Rotter and Bob Weinberg (Parada et al., 1984) and the second from Moshe Oren’s group (Eliyahu et al., 1984)), yet the Levine lab’s cDNA clone, despite being derived from a murine teratocarcinoma, showed absolutely no ability to score as collaborating oncogene. Coupled with the absence of p53 analogs encoded by transforming retroviruses, these findings conspired to bring p53’s oncogenic status into question and indeed to marginalize it as a potential cancer driver in comparison to myc, ras, and a host of other ‘classic’ oncogenes then grabbing the headlines at national and international oncogene meetings.

To this end, I joined the p53 team (or perhaps increased the body count sufficiently to now call it a team)—notably, Andrew Tan, a senior graduate student who had made numerous linker-insertion p53 mutants in effort to map the T antigen-binding domain in p53 and perform functional studies, and Cathy Finlay, a relatively new postdoc with superb skills in mammalian cell culture and rodent tumorigenesis studies resulting from her training at the Wistar Institute. Indeed, to a much greater degree than the specific experiments that followed and the rigorous and vigorous vetting of resultant data in lab meetings and elsewhere, it was Arnie’s expectation of productive teamwork that set the pace for our subsequent discoveries and for my emergence as a scientific thinker in my own right (I hope). Although Cathy Finlay and I formed a synergistic bond that enabled several discoveries over my time in the lab, there were many other generous souls in the Levine lab who shared Arnie’s love of discovery and who imparted this to me as well. To briefly scratch the surface of the Levine world of science: Alan Frey provided me with my first appreciation of immunology and more importantly taught me how to make antibodies in rabbits (Hinds et al., 1987), Terry van Dyke and Jeff Marks provided an appreciation of tumor modeling in mice (Marks et al., 1989), and collaboration with Cathy Clarke led to insights into the role of mutant p53’s then-mysterious partnering with hsc70 (Clarke et al., 1988). Each of these experiences enriched us all, I believe, and owed their success to the environment Arnie offered and expected.

While this type of intra-lab partnering hopefully occurs in many groups, I remain convinced that the teamwork encouraged by Arnie in partnering me with Cathy Finlay was unusually insightful. The initial studies of the transforming abilities of Andrew Tan’s mutants and subsequent comparison of the Levine lab’s cDNA clone (from which Andrew’s mutants were derived) and an ostensibly wild-type p53 genomic clone obtained from Moshe Oren constituted the bulk of my thesis work and were all done as a highly productive collaboration with Cathy Finlay. Ultimately, the publication of several papers focused on p53-mediated transformation and tumorigenesis culminated in the discovery that wild-type p53 suppresses and mutant p53 drives tumor formation (e.g. Finlay et al., 1989; Hinds et al., 1989). We have reviewed the events in the Levine lab and in the p53 field overall that led to these discoveries (Levine et al., 2004), but it is important to emphasize that none of this would have happened without countless meetings in the staff lounge adjacent to our lab in the LTL, where hypotheses, both sublime and ridiculous, were bandied about (as were libations of the red label and black label variety) and Arnie’s true passion—hashing out the data and letting it guide the process of discovery—enabled our mundane daily experiments to reveal fundamental truths about the process of oncogenesis. I am forever grateful for Arnie’s guidance in shaping my scientific mind, for his role in producing the amazing research environment in his lab and in the department, and for introducing me to the incredible synergies possible in partnering with brilliant lab mates like Cathy Finlay, Al Frey, Jeff Marks, Terry van Dyke, Cathy Clarke, Gigi Lozano, Father George Salzmann, and numerous others who helped guide me through the travails of graduate training. Arnie, here’s to 80, and I can only hope to accomplish a small fraction of what you have achieved when I’ve reached this milestone.
